# Exosomes and organ-specific metastasis

**DOI:** 10.1016/j.omtm.2021.05.016

**Published:** 2021-06-04

**Authors:** Lei Zhao, Xiaoting Ma, Jing Yu

**Affiliations:** 1 Cancer Center, Beijing Friendship Hospital, Capital Medical University, No. 95 Yong An Road, Xi Cheng District, Beijing 100050, China

**Keywords:** exosome, integrin, metastasis, organotropism, tumor

## Abstract

The transmission of information between tumor cells and other cell types in the tumor microenvironment plays an important role in tumor metastasis and is critically modulated by exosomes and other mediators. Tumor-derived exosomes can promote epithelial-mesenchymal transition, angiogenesis, immune escape, formation of the pre-metastatic microenvironment, and transmission of drug-resistant molecules, thereby promoting tumor growth, invasion, and metastasis. Integrins are important regulatory molecules on exosomes that can locate metastatic cells at the initial stage of metastasis and show good organotropism. This fact suggests that a clear understanding of the roles of exosomal integrins will be beneficial for future clinical applications. Follow-up studies on exosomes using continuously updated purification techniques and identification methods are extremely important. In addition to their potential as cancer biomarkers, exosomes also provide new research directions for precision medicine. Currently, exosomes have potential value in disease treatment and provide clinicians with more meaningful judgment standards.

## Graphical abstract

**Figure:**
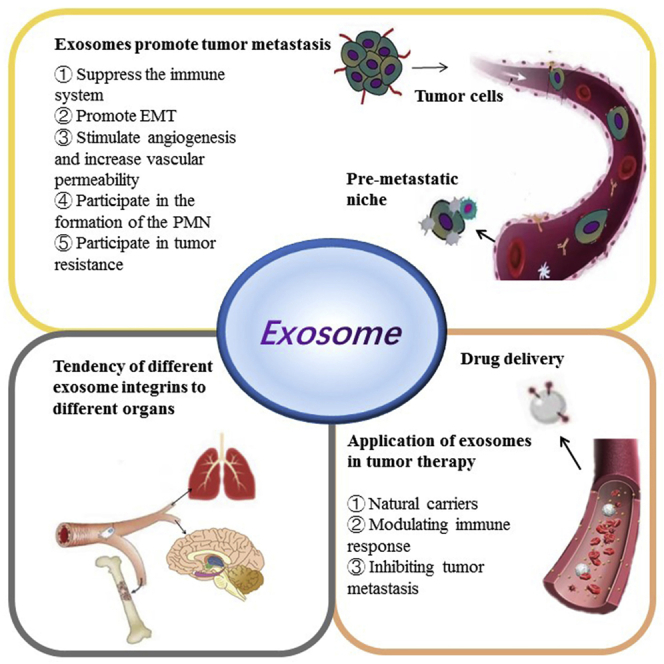

## Teaser

Exosomes are one of the important mediums of intercellular communication and play important roles in tumor metastasis. In this study, the multi-faceted role of exosomes in tumor metastasis suggests their potential value in tumor treatment.

## Introduction

Exosomes are the most widely studied of the three main subgroups of extracellular vesicles released from mammalian cells (the other two subgroups being microvesicles and apoptotic vesicles).^1^ Exosomes originate from multi-vesicular bodies (MVBs) and form granular nanoscale vesicles with a diameter of 30~150 nm by fusion, invagination, and budding with the membrane. They were first found in the supernatant of sheep red blood cells cultured *in vitro*.^1^^,^^2^ Almost all cells in the body, including tumor cells, can produce exosomes that carry a variety of proteins: messenger RNAs (mRNAs), microRNAs (miRNAs), long non-coding (lnc)RNAs, DNA, and lipids. The surface molecules of exosomes are primarily composed of integrins (ITGs), and transmembrane 4 superfamily tetraspanins. CD9, CD63, and CD81 are often used as specific exosomal markers.^3^^,^^4^ Exosomes are widely present in urine, blood, pleural and peritoneal effusions, saliva, bile, semen, and other body fluids but are secreted particularly by tumor cells, which may be related to Rab3D overexpression, Wnt pathway activation, and the acidic tumor microenvironment.^5^, ^6^, ^7^ Recent studies have shown that exosomes, which carry a large number of functional molecules, are a potentially therapeutically exploitable mode of intercellular signaling. By fusing with the target cell membrane, exosomes initiate intercellular communication and deliver functional molecules including miRNAs and proteins. Exosomes can participate in a series of processes such as immune responses, cell migration, proliferation, differentiation, and tumor invasion.

Although the roles of most substances in tumors are unclear, previous studies have shown that tumor-derived exosomes can induce epithelial-mesenchymal transition (EMT), promote angiogenesis and vascular permeability, establish a tumor pre-metastatic niche (PMN), and transmit drug-resistant molecules. Additionally, many exosome-derived tumor markers are widely used in clinical applications.For example, carcinoembryonic antigen (CEA) is significantly increased in digestive tract tumors, lung cancer, and breast cancer, and prostate-specific antigen (PSA) is significantly expressed in prostate cancer.^8^ One of the newly discovered carbonic anhydrase (CA) family isomers, CA IX, is a transmembrane glycoprotein composed of acidic amino acids that play an important role in regulating cell proliferation and transformation. CA IX is widely expressed in prostate cancer, lung cancer, renal clear cell carcinoma, and other malignant tumors.^9^^,^^10^ ITGs are important regulatory molecules on exosomes that interact with extracellular matrix (ECM) proteins and play a decisive role in organ tropism transfer.^11^ Exosomes can also carry endogenous or exogenous nucleic acid and protein molecules, which can regulate tumor cell proliferation, metastasis, and invasion after being ingested by target cells. This article will review how tumor-derived exosomes promote tumor metastasis, how this activity manifests in different tumor types, the roles of ITGs in organ-specific metastasis, methods of isolating and identifying exosomes, and finally, applications of exosomes for antitumor therapy.

## Exosomes promote tumor metastasis

### Exosomes suppress the immune system

**Figure 1:**
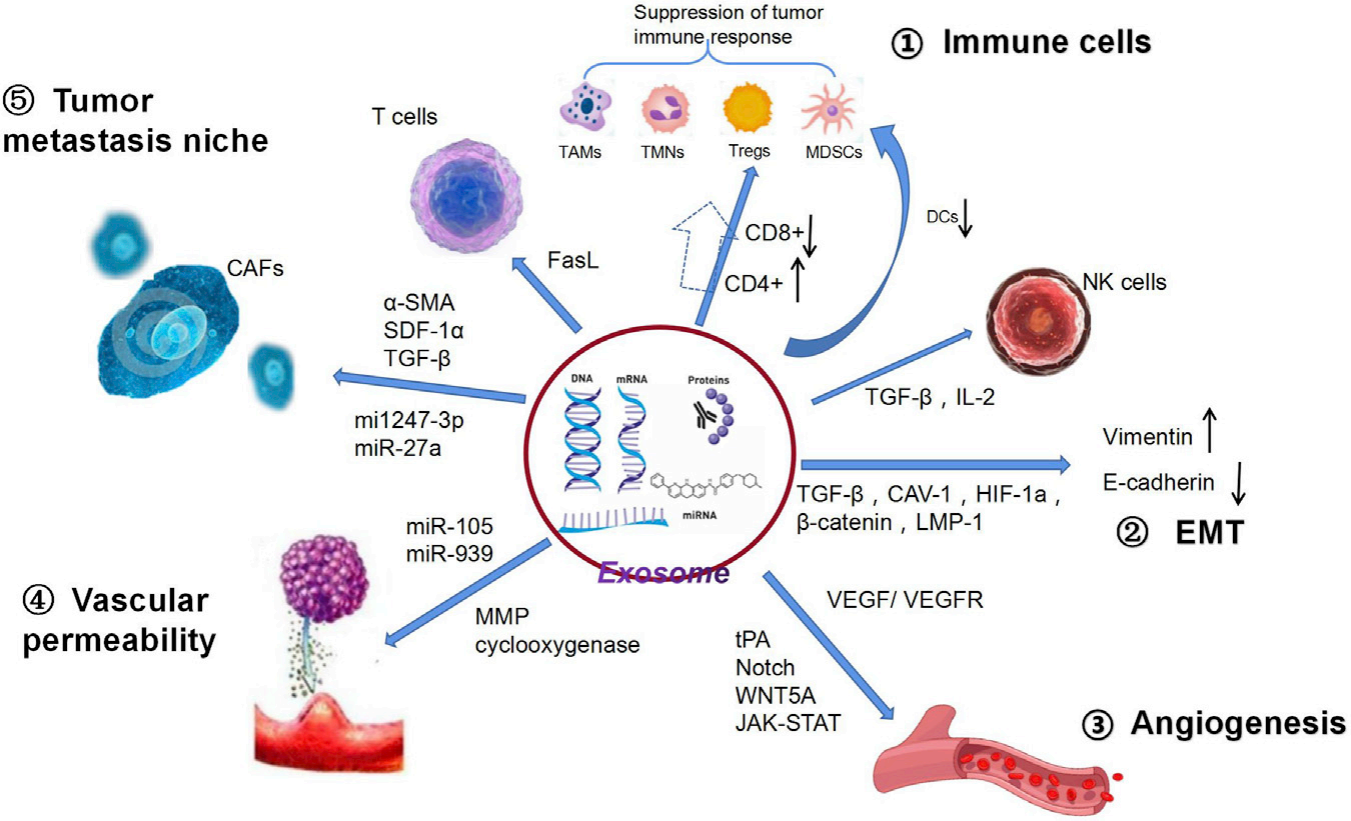
Exosomes promote the process of tumor metastasis Suppressing the immune system: exosomes recruit TAMs, TANs, Tregs, and MDSCs to distant secondary sites. FasL and other effector molecules can mediate T cell apoptosis. Exosomes can promote the conversion of helper T cells (CD4^+^) to Tregs and reduce cytotoxic T cells (CD8^+^). They can also weaken the ability of natural killer cells by secreting TGF-β or blocking IL-2 and further suppress MDSCs by inhibiting the proliferation and maturation of dendritic cells. Promoting EMT: TGF-β, CAV-1, HIF-1α, catenin β1, and LMP-1 in exosomes upregulate vimentin and downregulate E-cadherin. Stimulating angiogenesis: exosomes directly promote angiogenesis by activating the VEGF/VEGFR pathway or indirectly promote angiogenesis through the tPA-dependent pathway, Notch pathway, WNT5A pathway, and JAK-STAT pathway. Increasing vascular permeability: miR-105 and miR-939 in exosomes increase the permeability of blood vessels by destroying the transvascular endothelial barrier. MMP and COX can increase vascular permeability through vascular remodeling. Participating in the formation of PMN: SDF-1α, α-SMA, and TGF-β in exosomes can convert adipose-derived mesenchymal stem cells into CAFs. miR-27a and miR-1247-3p can promote CAF activation.

Tumor-derived exosomes play an important role in immune regulation and can cause the recruitment of suppressive immune cells. After exosomes upregulate the expression of proinflammatory factors, the local inflammatory microenvironment can induce tumor cells to produce chemokines and cytokines. These factors cooperate with tumor-generated exosomes to recruit tumor-associated macrophages (TAMs), tumor-associated neutrophils (TANs), regulatory T cells (Tregs), and myeloid-derived suppressor cells (MDSCs) to distant secondary sites, thereby suppressing antitumor immune responses (Figure 1).^12^, ^13^, ^14^

Some tumor-associated antigens carried by tumor-derived exosomes can stimulate immune cells to produce anti-tumor immune responses, but in more cases, exosomes interfere with immune recognition and inhibit the function of immune cells, such as T cells, dendritic cells (DCs), and natural killer (NK) cells, leading to immune escape of tumor cells, which promotes tumor development. Tumor-derived exosomes have been shown to induce loss of T cell activation. Fas ligand (FasL) and other effector molecules carried by exosomes mediate T cell apoptosis (Figure 1).^15^, ^16^, ^17^ Conversely, tumor-derived exosomes amplify Tregs through the transforming growth factor (TGF)-β1 and interleukin (IL)-10 pathways and increase the inhibitory activity of Tregs by upregulating FasL and other effector molecules.^18^ The upregulation of Tregs contributes to the ability of the tumor microenvironment to facilitate escape from immune responses.^15^ Studies have shown that melanomas release high levels of extracellular vesicles, mainly in the form of exosomes, which carry programmed death ligand 1 (PD-L1) on their surface. Interferon-γ (IFN-γ) can upregulate PD-L1 on these vesicles, thereby inhibiting the function of CD8^+^ T cells.^19^ Even in a model of anti-PD-L1 antibody resistance, removal of exosomal PD-L1 inhibits tumor growth.^20^ Therefore, the level of exosomal PD-L1 can modulate the dynamic interactions between tumor and immune cells. Tumor-derived exosomes can also weaken the activity of NK cells by secreting TGF-β or blocking IL-2.^21^ Finally, tumor-derived exosomes can inhibit the proliferation and maturation of DCs and enhance the inhibitory effect of MDSCs, thereby helping tumor cells escape immune surveillance (Figure 1).^22^^,^^23^

### Exosomes promote EMT

EMT is a physiological or pathological process in which epithelial cells are transformed into a mesenchymal phenotype through a specific process.^22^ Proteins, miRNAs, and other contents of tumor-derived exosomes can induce EMT in tumor cells, weaken the tight junctions and adhesive connections of cells, and enhance cell migration, thus promoting tumor metastasis. Tumor exosomes are rich in TGF-β, caveolin-1 (CAV-1), hypoxia-inducible factor (HIF-1α), catenin β1, and latent membrane protein (LMP-1), which can upregulate the mesenchymal marker vimentin and downregulate the epithelial marker E-cadherin (Figure 1).^22^^,^^25^ Aga et al.^26^ further confirmed that LMP-1 significantly increased HIF-1α levels in exosomes and increased the invasion and metastasis of nasopharyngeal carcinoma through the EMT process. Xiao et al.^27^ found that after co-culturing normal human epidermal melanocytes and melanoma cell-derived exosomes, E-cadherin was downregulated, and vimentin was simultaneously significantly increased in normal cells. Franzen et al.^25^ found that exosomes isolated from bladder urothelial tumor cells induced obvious EMT characteristics after acting on bladder transitional cells, including downregulating E-cadherin and upregulating vimentin, SNAIL, and TWIST. Additionally, the miRNAs within exosomes also play key roles in inducing EMT. miRNA (miR)-23a promotes EMT in lung and gastric cancer cells by inhibiting the expression of E-cadherin, whereas miR-193a-3p, miR-210-3p, and miR-5100 promote cancer cell invasion by activating EMT through the signal transducer and activator of transcription 3 (STAT3) pathway.^29^, ^30^, ^31^

### Exosomes stimulate angiogenesis and increase vascular permeability

Tumor-derived exosomes containing proteins, miRNAs, and lncRNAs can directly promote angiogenesis by activating the vascular endothelial (VE) growth factor (VEGF)/VEGF receptor (VEGFR) pathway or indirectly promote angiogenesis by the tissue plasminogen activator (tPA)-dependent pathway, Notch pathway, WNT5A pathway, and Janus kinase (JAK)-STAT pathway, which play important roles in cancer development (Figure 1).^32^ Proteins such as epidermal growth factor receptor type III (EGFRvIII) and CD147 in tumor-derived exosomes can contribute to angiogenesis through VEGF/VEGFR and its downstream pathways.^33^^,^^34^ VEGF exists in exosomes of cells from ovarian cancer, multiple myeloma, and nasopharyngeal cancer and promotes angiogenesis by binding to VEGFR of endothelial cells.^35^ Additionally, CD147 expression in exosomes of ovarian cancer cells can enhance tumor angiogenesis by increasing the secretion of VEGF and matrix metalloproteinases (MMPs).^34^ DeRita et al.^36^ confirmed that exosomes from prostate cancer cells were rich in the steroid receptor co-activator, which was speculated to enhance angiogenesis by upregulating VEGF. Annexin A2 in exosomes secreted by human breast cancer cells increases plasminogen production through the tPA-dependent pathway, thereby stimulating angiogenesis.^32^ *In vivo* experiments have confirmed that tumor-derived exosomes can promote angiogenesis via the Notch ligand delta-like 4.^37^ Additionally, Lang et al.^38^ showed that glioma cell exosomes contain lncRNAs, including lncCCAT2 and lncPOU3F3, which can stimulate angiogenesis by upregulating VEGF and other factors. Exosomes secreted by colorectal cancer (CRC) cells are rich in miR-25-3p, which increases angiogenesis and loosens the connections between VE cells by targeting the transcription factors Krüppel-like factor (KLF)2 and KLF4.^39^

In addition to participating in angiogenesis, tumor exosomes can also increase vascular permeability to promote the formation of PMN.^40^ Peinado et al.^41^ found that exosomes from the highly malignant melanoma cell line B16-F10 enhanced the permeability of lung endothelial cells in mice compared with non-metastatic cell lines. The miR-105 and miR-939 secreted by metastatic breast cancer cells destroy the VE barrier in the metastatic pathway, thereby increasing vascular permeability and promoting distant metastasis.^42^^,^^43^ Breast cancer cells can also express MMPs and cyclooxygenase (COX), which promote vascular remodeling and vascular permeability and thereby accelerate metastasis (Figure 1).^44^ Studies have shown that exosomes released by hypoxic tumors are more likely to promote angiogenesis and increase vascular permeability.^1^

### Exosomes participate in the formation of the PMN

Recent studies have shown that stromal cells receive tumor-derived exosomes that promote the formation of a PMN.^1^ Tumor-derived exosomes can promote the formation of cancer-associated fibroblasts (CAFs), which can be activated by normal fibroblasts in the primary tumor site or in metastatic tissues or can be transformed from mesenchymal stem cells, adipocytes, and endothelial cells.^45^^,^^46^ TGF-β in exosomes from gastric cancer, bladder cancer, prostate cancer, or breast cancer can promote the formation of CAFs by activating the SMAD pathway. Ovarian cancer-derived exosomes express α-smooth muscle actin (α-SMA), stromal cell-derived factor (SDF)-(1α), and TGF-β, which can transform adipose-derived mesenchymal stem cells into CAFs.^47^ LMP-1 is a major viral oncogene that is expressed in most Epstein-Barr (EB) virus-related cancers.^48^^,^^49^ Nkosi et al.^49^ showed that LMP-1-modified extracellular vesicles can reshape the tumor microenvironment by changing the expression of different target genes including cadherin, MMP9, MMP2, and ITG α5. Wu et al.^50^ also found that LMP-1-modified extracellular vesicles promoted tumor proliferation and tumor PMN formation by activating CAFs. Finally, the miRNAs carried by tumor-derived exosomes are also critical for CAF formation. For example, miR-27a in exosomes derived from gastric cancer cells and miR-1247-3p in exosomes derived from liver cancer cells can both further activate CAFs (Figure 1).^51^^,^^52^

Macrophage inhibitory factor (MIF) and TGF-β in exosomes derived from pancreatic cancer cells can promote hepatic stellate cells to secrete abundant fibronectin and recruit bone marrow-derived macrophages and neutrophils to transform the liver into a suitable microenvironment for colonization by pancreatic cancer cells.^53^ Annexin A2 released by breast cancer cell-derived exosomes can induce macrophage-mediated activation of the p38 mitogen-activated protein kinase (MAPK), nuclear factor κB (NF-κB), and STAT3 pathways and increases the secretion of IL-6 and tumor necrosis factor (TNF)-α, thereby contributing to the formation of a premetastatic inflammatory microenvironment in distant organs such as the lung and brain.^32^ Many miRNAs in exosomes derived from prostate cancer cells target the BMPR2 and HNRNPU genes, which are related to osteoblast differentiation, and thus participate in PMN formation.^54^ Peinado et al.^41^ found that when exosomes secreted by B16-F10 cells reached the lungs of mice, levels in lung tissues of heat shock proteins (HSPs), S100a8 and S100a9, which are all related to PMN formation, increased compared with ordinary melanocyte exosomes.

### Exosomes are involved in the drug-resistance mechanism of tumors

Several studies have found that disorders in tumor-related miRNAs, proteins, and signal transduction pathways are related to tumor chemotherapy resistance.^55^^,^^56^ First, the acidic tumor microenvironments play a major role in the drug resistance, proliferation, and metastasis of malignant tumors. Studies have shown that tumor cells have the ability to survive in a hypoxic/acidic environment, which weakens uptake of weakly alkaline chemotherapeutic drugs, and thus compromises their efficacy.^57^ Further studies showed that low pH can also increase the ability of tumor cells to release exosomes.^57^ Tumor cells secrete exosomes carrying drug resistance-related molecules, and through exosomes, they interact in the tumor microenvironment to transfer drug-resistant molecules, thus increasing the tolerance of tumor cells to drugs. At the same time, tumor cell exosomes can also participate in drug efflux, thus affecting the effective blood drug concentration and ultimately promoting drug resistance in tumor cells. In a study of breast cancer cells resistant to Adriamycin, Yu et al.^58^ found that miR-222 expression in drug-resistant strains was significantly higher than in sensitive strains. Further studies have confirmed that breast cancer cell-derived exosomes can deliver miR-222 to regulate drug resistance in tumor cells, thereby mediating resistance in the sensitive breast cancer cell line MCF-7.^58^ Xiao et al.^59^ confirmed that significantly more exosomes were released by A549 lung cancer cells after exposure to cisplatin (DDP); furthermore, the exosomes released by A549 cells under DDP exposure can reduce the sensitivity of other A549 cells to DDP. Qin et al.^60^ found that miR-100-5p can alter the sensitivity of A549 cells to DDP by regulating expression of the mammalian target of rapamycin (mTOR) gene.

Although the detailed mechanistic link between tumor cell resistance and exosomes is not yet fully understood, the above-mentioned studies provide new ideas for future exploration of tumor resistance.

## Relevant research into the roles of exosomes during metastases in various solid tumors

### Role of exosomes in breast cancer metastasis

Previous studies have confirmed the role of breast cancer cell-derived exosomes in metastasis. As reported, decreased sirtuin 1 (SIRT1) expression is associated with the metastatic spread of breast cancer cells.^61^ Knocking down SIRT1, the most widely studied member of the NAD^+^-dependent deacylase family, can destabilize the mRNA encoded by the A subunit, which encodes the lysosomal vacuolar H^+^-ATPase (V-ATPase) proton pump (ATP6V1A), resulting in less protein expression. The decreased ATP6V1A levels diminish lysosomal degradation activity, which causes there to be more MVBs. These MVBs fuse with the plasma membrane to release exosomes containing different carriers, which strongly promotes the growth and migration of tumor cells. Furthermore, following the downregulation of SIRT1, there is significantly increased secretion of cathepsin, which degrades ECM and allows tumor cells to invade the surrounding tissues, which ultimately promotes breast cancer metastasis.^61^ Additionally, miR-105 downregulates tight junction proteins, destroying tight intercellular connections and the natural barrier function, thereby inducing cell migration.^42^ miR-9 and miR-155 induce cell migration by downregulating phosphate and tension homology deleted on chromsome ten (PTEN) and dual-specific protein phosphatase 14 in recipient cells.^62^ CAV-1 in breast cancer exosomes can promote cell migration and invasion *in vitro*.^63^

Recent evidence indicates that ITG α6β4 and αvβ3 on the surface of breast cancer exosomes increases lung metastasis.^11^ In terms of immunity, the proliferation of breast cancer exosomes can stimulate macrophage polarization to create favorable conditions for lymph node metastasis (LNM).^64^ Annexin A2 from breast cancer exosomes can stimulate the secretion of IL-6 and TNF-α by inducing macrophage-mediated p38 MAPKs.^32^ As mentioned above, miR-105 and miR-939 in breast cancer exosomes, as well as MMP and COX, can increase vascular permeability and promote distant metastasis (Table 1).^42^, ^43^, ^44^

**Table 1:** The main exosomes derived from various solid tumors

	Protein	MicroRNA (miR)
Breast cancer	Annexin A2^32^	miR-105/miR-939^42^^,^^43^
	stimulating angiogenesis and promoting the secretion of interleukin-6 (IL-6) and TNF-α	increasing vascular permeability
	CAV-1^63^	miR-9/miR-155^62^
	promoting cell migration and invasion	promoting cell migration and invasion
	MMP/COX^44^	
	increasing vascular permeability	
Prostate cancer	Hyal1^68^	miR-940^65^
	promoting the mobility of mesenchymal cells	promoting the formation of PMN
	TF/PAI-1^70^	miR-21-5p/miR-139-5p^71^
	promoting cell invasion and angiogenesis	promoting the formation of PMN
	TGF-β1^69^	
	promoting tumor growth and angiogenesis	
Melanoma	Rab27A^67^	miR-155/miR-210^74^
	promoting the metastasis by changing the ability of surrounding cells to invade and move	promoting the formation of PMN
Lung cancer	Rab3D^75^	miR-21/miR-29a/miR-205-5p/miR-200b^77^^,^^78^
	facilitating the EMT process	promoting tumor growth and metastasis
	TGF-β1^76^	miR-210/miR-21/miR-9^81^^,^^82^
	facilitating the EMT process and increasing tumor cell invasion	promoting angiogenesis
	IL-10^76^	
	increasing tumor cell invasion	
	EGFR^79^	
	inhibiting the anti-tumor function of CD8^+^ T cells	
	MICA/B^77^	
	inhibiting lymphocyte function	
Colon cancer	FasL^87^	miR-21^84^^,^^85^
	promoting T cell apoptosis	inhibiting cell apoptosis
		miR-25-3p^39^
		destroying the tight connections of vascular endothelial cells and promoting angiogenesis
Liver cancer	14-3-3ζ^97^	miR-1247- 3p^52^
	inhibiting the anti-tumor function of infiltrating T cells	promoting activation of CAF
		miR-93^94^
		promoting the proliferation and metastasis of tumor cells
		miR-210-3p^98^
		promoting angiogenesis
		miR-103^99^
		destroying the integrity of the endothelial cell adhesion connection
Pancreatic cancer	MIF/TGF-β^53^	
	activating the fibrotic pathway and promoting the formation of PMN	
Ovarian cancer	Alix/TSG101/Rab/annexin/CD9/CD82/CD63/CD81/Hsp90/Hsc70/MHCI/II/Nanog/TLR^4^	miR-99a-5p^103^
	promoting the proliferation and metastasis of tumor cells	promoting cell invasion by upregulating fibronectin and hyaline
	CD147/ATF2/MTA1^34^^,^^108^	
	promoting angiogenesis	
	FasL	
	promoting T cell apoptosis	

ATF2, activating transcription factor 2; MTA1, metastasis-associated protein 1; TF/PAI-1, tissue factor/plasminogen activator inhibitor 1.

### Role of exosomes in prostate cancer metastasis

The invasion and metastasis process of prostate cancer is roughly divided into three stages. The first stage is blood vessel invasion, and exosomes from prostate cancer cells are first absorbed by the surrounding prostate epithelial cells, inducing EMT and promoting ECM degradation and remodeling. This improves the ability of vital prostate cancer cells to invade blood vessels. Additionally, exosomes mediate increased vascular permeability by damaging the barrier, making prostate cancer cells more likely to invade blood vessels. The second stage is circulation of prostate cancer cells to the bone marrow. During this process, exosomes contribute to the osteophilicity of circulating prostate cancer cells, facilitating the spread of prostate cancer cells to the bone marrow. Hashimoto et al.^65^ found that a large amount of Human serum albumin (HSA)-miR-940 is secreted by prostate cancer cells. HSA-miR-940 promotes the osteogenic differentiation of human mesenchymal stem cells *in vitro* and induces extensive osteogenic lesions in the bone metastatic microenvironment *in vivo*.^65^ The third stage is altering the bone marrow microenvironment. Exosomes can promote formation of the PMN by “cultivating” bone marrow precursor cells and immune regulators and prepare for the environment, materials, and other aspects of colonization by prostate cancer cells. ITG αvβ3, αvβ6, α4β1, and αvβ3 all have a tropism effect on bones.^66^^,^^67^

McAtee et al.^68^ found that prostate cancer exosomes containing Hyal1 can enhance the migratory ability of interstitial cells through the focal adhesion kinase (FAK)-ITG pathway. Moreover, prostate cancer exosomes can activate TGF-β1-dependent fibroblast differentiation to the myofibroblast phenotype and promote tumor growth and angiogenesis, but the direct use of soluble TGF-β1 cannot achieve these cancer-promoting effects. Hence, the role of prostate cancer exosomes in EMT is very important.^69^ Prostate cancer exosomes highly expressing mutant EGFRvIII are rich in tissue factor and plasminogen activator inhibitor, which can be activated by protease activator receptor-1 to increase tumor cell invasiveness and angiogenesis.^70^ Bone marrow receptor cells absorb prostate cancer exosomes, inducing expression of several inflammatory mediators (S100 proteins, TGF-β, IL-6, IL-8, and TNF-α). This then activates and remodels interstitial cells and recruits bone marrow-derived cells (BMDCs) to the PMN, which jointly promote tumor progression.^54^ At the same time, the differentially expressed miRNAs (e.g., miR-21-5p and miR-139-5p) in prostate cancer exosomes also regulate PMN formation (Table 1).^54^ After receiving exosomes, bone marrow fibroblasts differentiate into myofibroblast-like cells through the TGF-β/SMAD pathway, enhancing the recruitment of BMDCs and stimulating tumor colonization and growth in bone marrow.^72^

### Role of exosomes in melanoma metastasis

Studies have shown that compared with normal skin or moles, the Ras-related protein Rab-27A (RAB27A) is upregulated in melanoma and is related to the stage of the lesion. After knocking out RAB27A, RAB27A-rich exosomes can change the invasion phenotype of melanoma cells, which indicates that exosomes can promote melanoma metastasis by altering the invasion and motility of melanoma cells.^67^ As mentioned above, after co-cultivating normal human epidermal melanoma cell lines with exosomes derived from melanoma cells, E-cadherin was significantly downregulated compared with normal cell epithelial cells, whereas the mesenchymal marker vimentin was significantly upregulated.^27^

Exosomes derived from melanoma cells can promote the accumulation of MDSCs, directly inhibit the function of T cells and NK cells in the lung and liver of mice, and impair DC maturation in lymph nodes.^73^ Exosomal PD-L1 levels are positively correlated with the degree of metastasis in melanoma patients, indicating that this exosome-mediated immunosuppressive mechanism plays an important role in promoting metastasis.^19^ In addition to enhancing lung endothelial permeability, melanoma-derived exosomes also induce vascular leakage and reprogram bone marrow progenitor cells into a c-Kit^+^ Tie2^+^ Met^+^ pro-angiogenic phenotype.^41^ It was observed by tracing exosomes that 1833-BoT and 4175-LuT promoted pulmonary vascular leakage after injection.^11^ Additionally, exosomes derived from malignant melanoma can coordinate the formation of PMN by guiding BMDCs to the anterior metastatic phenotype and promoting their mobilization to future metastatic sites including lymph nodes and lungs.^33^ Melanoma-derived exosomal miR-155 and miR-210 can also reprogram human adult dermal fibroblasts (HADF) and cause extracellular acidification, which contributes to the production of PMN, thereby promoting metastasis (Table 1).^74^ Moreover, a small nuclear RNA carried by melanoma-derived exosomes activates Toll-like receptor (TLR)3, which leads to the recruitment of metastatic neutrophils to lung PMN.^73^ At the same time, expression of the HSPs, S100a8 and S100a9, in lung tissues increase. Both proteins are closely related to the formation of PMN.^41^

### Role of exosomes in lung cancer metastasis

First, highly metastatic lung cancer exosomes can induce normal bronchial epithelial cells to express vimentin, driving the EMT cascade in these cells, which endows recipient cells with increased migration, invasion, and proliferation capabilities. Rab3D protein and TGF-β, released by exosomes derived from the lung adenocarcinoma cell line A549, promote EMT through different pathways, thereby enhancing lung cancer invasion and metastasis.^75^ Second, TGF-β and IL-10, which are closely related to tumor cell invasion, are increased in the exosomes of metastatic small cell lung cancer cells, and this activates SMAD, phosphatidylinositol 3-kinase (PI3K)/AKT, BRAF-MAPK, and other signaling pathways, thereby promoting tumor metastasis.^76^ Moreover, miR-21 and miR-29a in lung cancer exosomes can bind to TLRs and activate the NF-κB pathway, leading to tumor growth and metastasis.^77^ Lin et al.^78^ found that miR-205-5p and miR-200b were upregulated in serum-derived exosomes of lung cancer patients. Knocking out or silencing these miRNAs inhibits the growth and invasion of lung cancer cells.

Lung cancer cell-derived exosomes participate in immune escape and promote the occurrence and development of lung cancer. Huang et al.^79^ found that EGFR within lung cancer exosomes can induce immune-tolerant DCs and then generate specific Tregs. These Tregs can inhibit the anti-tumor function of CD8^+^ T cells, thereby affecting the development of lung cancer. Ligands in exosomes, such as major histocompatibility complex (MHC) class I chain-related protein (MIC)A and MICB, downregulate NKG2D receptors by binding to them and inhibiting lymphocyte function, eventually leading to immune escape of tumor cells.^77^ In terms of angiogenesis, miR-210 released by lung cancer exosomes inhibits specific target genes by secreting neutral sphingomyelinase 2, thereby promoting tumor angiogenesis.^80^ Experimentally, miR-210 can promote tumor angiogenesis by downregulating tyrosine receptor kinase A3 in endothelial cells.^81^ In addition, miR-21 and miR-9 also promote angiogenesis in lung cancer (Table 1).^82^

### Role of exosomes in colon cancer metastasis

Recently, various cell lines have been used to study CRC exosomes, such as LIM1215, LIM1863, HCT-29, SW480, and WiDr. The contents of exosomes derived from these cell lines are similar to some extent. Experimentally, high miR-21 levels were detected in exosomes derived from HCT-29, SW480, and WiDr cells.^2^^,^^83^ miR-21 is the most common and highly upregulated miRNA in CRC cell lines.^2^ Overexpression of miR-21 can regulate the expression of invasion- and metastasis-related target genes in the hepatocellular carcinoma (HCC) cell line HepG2 and the lung cancer cell line A549 by inhibiting the expression of programmed cell death 4 and PTEN, which are involved in apoptosis.^84^^,^^85^ Exosomes derived from SW480 cells can also be absorbed into HepG2 receptor cells through dynamic dependent endocytosis. Once internalized, the exosomes localize in lysosomes, which in turn induce the regulated phosphorylation of extracellular regulatory protein kinases 1/2 (ERK1/2) and initiate cancer cell migration by activating the MAPK pathway.^86^ Finally, natural antisense RNAs in CRC exosomes may promote malignant growth of liver and lung tumors by regulating the expression of target genes (e.g., MDM2 and CDKN1A) in the cytoplasm of HepG2 and A549 cells.^2^

FasL in CRC exosomes can downregulate the expression of the surface T cell receptor (TCR) and promote T cell apoptosis.^87^ CRC exosomes carrying miR-21 can activate TLR7 in the cytoplasm of liver macrophages, and the activated macrophages secrete inflammatory cytokines (IL-6, S100A, and MMP) that promote liver metastasis. In turn, the upregulated IL-6 can further stimulate miR-21 expression.^88^^,^^89^ In terms of angiogenesis, miR-25-3p in CRC exosomes can destroy the tight connections of VE cells and promote angiogenesis by targeting the transcription factors KLF2 and KLF4 (Table 1).^39^

### Role of exosomes in liver cancer metastasis

Liver cancer cells, adipocytes, fibroblasts, immune cells, and other cells form a complex liver cancer microenvironment, and exosomes serve as the medium for cell communication and are responsible for the transmission of information among these cells. *In vitro* experiments have shown that exosomes derived from HepG2 cells can activate several phosphokinases and the NF-κB pathway in adipocytes and upregulate related inflammatory factors.^90^^,^^91^ Adipocytes treated with HepG2 exosomes during co-culture with liver cancer cells showed that these exosomes promote the proliferation and metastasis of liver cancer cells. HepG2 exosomes can induce the differentiation of adipose tissue-derived mesenchymal stem cells into CAFs, which in turn promote the proliferation, migration, and invasion of HepG2 cells.^92^ Additionally, miR-1247-3p from liver cancer exosomes can activate CAFs.^52^ Chen et al.^93^ showed that exosomes derived from the highly metastatic hepatoma cell line MHCC97H activated the MAPK/ERK pathway, which induced EMT in moderately metastatic hepatoma cell lines and improved their metastatic ability. miR-93, lncRNA-HULC, and lncRNA-FAL1 in HCC exosomes can activate or inhibit the corresponding signaling pathways by regulating target protein expression in receiving cells, thereby enhancing tumor proliferation and invasion.^94^, ^95^, ^96^

For immune cells, liver cancer exosomes can deliver 14-3-3ζ protein to tumor-infiltrating T lymphocytes, inhibiting their anti-tumor function.^97^ In terms of angiogenesis, exosomes can deliver miR-210-3p from liver cancer cells to endothelial cells, directly targeting SMAD4 and STAT6 and enhancing angiogenic capacity.^98^ Additionally, miR-103 in HCC exosomes can target and regulate adhesion-related factors (e.g., VE-cadherin [VE-Cad], p120, and ZO-1) in VE cells, destroying the integrity of cell adhesion and promoting invasion and distant metastasis of HCC (Table 1).^99^

### Role of exosomes in pancreatic cancer metastasis

Pancreatic cancer cell-derived exosomes play key roles in activating the liver PMN. First, ITG αvβ5 on the surface of pancreatic cancer exosomes has a tropism effect on the liver. Second, macrophage MIF, an important component of pancreatic cancer exosomes, promotes fibrotic cytokine secretion after fusion with Kupffer cells, activates fibrotic pathways, and ultimately establishes a pro-inflammatory environment for metastasis. TGF-β can activate hepatic stellate cells and promote fibronectin secretion. These fibronectin deposits in the liver form a fibrotic microenvironment, which is conducive to the recruitment of BMDCs (macrophages and neutrophils) and leads to the formation of a PMN before liver metastasis.^53^ Studies have confirmed that inhibiting MIF can prevent all subsequent steps in PMN formation and therefore can prevent liver metastasis by pancreatic cancer cells (Table 1).^53^

### Role of exosomes in ovarian cancer metastasis

Unlike other human tumors, ovarian cancer preferentially invades the peritoneal cavity through ascites, which facilitates the involvement of various internal organs in the compartment.^4^ Exosomes can be isolated from the ascites and serum of ovarian cancer patients.^4^ These exosomes contain proteins unique to ovarian cancer, such as membrane proteins (Alix and TSG101), small GTPases (Rab proteins), annexin, transmembrane proteins (CD9, CD82, CD63, and CD81), HSPs (Hsp90 and Hsc70), antigens (MHC class I and class II), Nanog, and enzymes (phosphate isomerase, peroxidase, aldehyde reductase, and fatty acid synthase).^4^ These exosomes promote the metastasis of ovarian tumors. For example, Nanog, a transcriptional regulator, participates in the proliferation of tumor cells and the self-renewal of tumor stem cells. Nanog expression is significantly higher in exosomes extracted from ascites of high-grade serous ovarian cancer compared with benign peritoneal fluid, and the migration and invasion of ovarian cancer cells decrease when Nanog is knocked out.^100^, ^101^, ^102^ miR-99a-5p levels are significantly increased in ovarian cancer exosomes, and through upregulation of fibronectin and vitronectin, human peritoneal mesothelial cells promote cell invasion.^103^ Ovarian cancer exosomes can also facilitate the proliferation and invasion of tumor cells by promoting the transformation of host cells into TAMs and CAFs. Experimental evidence indicates that exosome-induced TAMs secrete abundant EGF, which activates EGFR signaling in surrounding ovarian cancer cells. This EGFR signal upregulates VEGF-C, which in turn upregulates intercellular adhesion molecule (ICAM)-1, thereby inducing tumor proliferation, migration, adhesion, and peritoneal implantation.^104^

Exosomes isolated from ovarian cancer ascites can block T cell function. GD3 is a ganglioside expressed on the surface of ascites exosomes that blocks T cells by acting on TCRs.^105^ Through TLR activation, ovarian cancer exosomes can also induce IL-6 production in monocytes, which in turn activates the STAT3 pathway in immune cells, stromal cells, and tumor cells, thereby supporting immune escape of cancer cells.^106^ Furthermore, ovarian cancer cells also release FasL-carrying exosomes, which downregulate surface TCR expression and promote T cell apoptosis.^4^ The NKG2D receptor, also a target for downregulation by ovarian cancer exosomes, inhibits the activity of NK cells.^107^ Regarding angiogenesis, ovarian cancer exosomes enhance the activity and migration of human umbilical vein endothelial cells.^108^ In addition to the increased secretion of VEGF and MMP through CD147 expression, proteomics has revealed that activated transcription factor 2 and metastasis-associated protein 1 in ovarian cancer exosomes can increase tumor angiogenesis (Table 1).^32^^,^^108^

## Role of exosome ITGs in organ-specific metastasis

Since the proposal of the “seed and soil” theory by Paget in 1889, the organ tendency of tumor migration has been well known. Accordingly, much research has focused on determining the internal determinants of organ-specific metastasis. Accumulating evidence proves that although tumor cells can reach all vascular-rich organs, metastatic colonization only succeeds in certain organs. Our study of tumor-derived exosomes in tumor metastasis has revealed the important position of exosome ITGs in organ-specific metastasis. Research on exosome ITGs in non-small cell lung cancer also confirms the role of ITGs in tumorigenesis and development. The first ITG antagonist cilengitide has been used in clinical trials in combination with chemotherapy drugs.^66^

### ITGs

ITGs are important regulatory molecules on exosomes that are heterodimers of α and β subunits connected by disulfide bonds. They interact with ECM proteins and are primarily involved in cell adhesion.^66^^,^^109^ The spherical head of each ITG serves as a linker for the ECM, whereas the C-terminal tail of the two subunits anchors to the intracellular actin cytoskeleton.^109^ All exosomes can express α2β1 and transfer to different organs according to the composition ratio.^11^ In vertebrates, the ITG family contains 18 α subunits and eight β subunits, which can be assembled into 24 heterodimers with various ligand-binding properties. Although many ligands bind to ITG receptors, non-collagen matrix proteins that contain the arginine-glycine-aspartic acid (RGD) sequence, including fibulin, laminin, and hyaline, are the major extracellular ligands of ITGs.^109^ Hence, ITGs bind intracellular and extracellular proteins and have unique bidirectional signal transduction properties.^109^

In recent years, there have been many studies on exosome ITG-related phenotypes. For example, α6β4 and α6β1 preferentially guide circulating melanoma cells to lungs,^110^ and α6β4 and αvβ5 can induce the metastasis of tumor cells to the lung and liver,^11^ respectively. In the breast cancer sub-cell line MDA-MB-231, 4175-LuT exosomes are preferentially localized in lungs, whereas 831-BrT exosomes are effectively localized in brains. Although exosomes from MDA-MB-231 are similar in size and morphology, their biological distributions are different, which further confirms the role of exosome ITGs in targeting different organs.^11^

### ITGs and LNM

As previously reported, α4 ITG, which is important for tumor LNM, is associated with carcinogenicity and LNM in various malignant tumors, including colon cancer, lung cancer, pancreatic ductal cancer, and melanoma.^111^, ^112^, ^113^ The α4 subunit can dimerize with the β1 and β7 subunits to form α4β1 and α4β7, respectively.^110^ In certain tumor cells, α4β1 is expressed on the cell surface and binds to vascular cell adhesion molecule 1 (VCAM-1) to promote attachment to lymphatic endothelial cells (LECs);^114^ however, α4β1 is also expressed on LECs in lymph nodes and after activation, plays an important role in capturing VCAM-1-positive tumor cells.^115^ Experimentally, inhibiting ITG α4β1 on LECs significantly prevents peri-tumor lymphangiogenesis and LNM (Table 2).^113^

**Table 2:** Exosome integrins and their targeted distribution organs

Target organ	Exosomal integrins
Lymph node	α4β1
	α4β7
Lung	αvβ3
	α6β4
	α6β1
	αvβ5
Liver	α2β1
	αvβ5
	α5β1
Brain	αvβ3
	αvβ5
	αvβ8
Bone	αvβ6
	αvβ3
	α4β1

### ITGs and lung metastasis

In preclinical *in vivo* breast cancer models, expression of αvβ3, a receptor for vitronectin and fibronectin, specifically guides tumor cells to the lung and promotes spontaneous breast-to-lung metastasis.^110^ However, αvβ3 cannot promote the proliferation of breast cancer cells *in vitro* or *in vivo*, indicating that αvβ3 may enhance the adhesion of tumor cells to pulmonary blood vessels but does not promote their proliferation.^116^ Furthermore, a successful melanoma cell lung metastasis model validated that αvβ3 is pivotal for guiding melanoma cells to the lungs. MK-0429, an αvβ3 inhibitor, significantly reduced the lung metastasis rate after injection into the tail vein.^117^

Hoshino et al.^11^ showed that exosomes expressing ITG α6β4 and α6β1, which are abundant in pulmonary exosomes, can interact with S100A4-positive fibroblasts and surfactant protein C-positive epithelial cells in the laminin-rich lung microenvironment.^118^ To verify these conclusions, Hoshino et al.^11^ used a short hairpin RNA to knock down ITG β4 in breast cancer 4175-LuT exosomes and found that the β4KD-labeled exosomes in the lungs were reduced by more than 3-fold compared with control 4175-LuT exosomes. Additionally, ITG αvβ5, especially the β5 subunit, alters vascular permeability of the lungs by regulating VEGF and TGF-β (Table 2).^119^

### ITGs and liver metastasis

ITG β1 promotes interactions between liver cells and the liver ECM to facilitate the proliferation and migration of fibroblasts, which contributes to liver fibrosis, a process that is closely related to the formation of liver PMN.^120^^,^^121^ Furthermore, ITG α2 primarily mediates the occurrence of liver metastasis by binding to type IV collagen, which is highly present in the liver sinus.^122^ An analysis of preclinical melanoma and breast cancer models confirmed the view that α2β1 activated by VE-cadherin can promote the occurrence of liver metastasis.^123^

Hoshino et al.^11^ showed that ITG αvβ5 primarily exists in hepatotropic exosomes and when expressed by pancreatic tumor exosomes, co-localizes with F4/80^+^ macrophages in the fibronectin-rich liver, which can specifically bind Kupffer cells and increase liver metastasis. Similarly, knocking down ITG β5 in pancreatic cancer BXPC-3-LIT exosomes resulted in a 7-fold reduction in liver uptake compared with control BxPC-3-LiT exosomes.^11^

ITG α5β1 is the only known α5 ITG that is an upstream regulator of c-Met, Src, and FAK.^110^ Inhibiting α5β1 was confirmed to decelerate liver metastasis in mouse models of ovarian cancer and CRC (Table 2).^124^^,^^125^

### ITGs and brain metastasis

Due to the blood-brain barrier, brain metastasis has always been a problem when treating advanced tumors. The αv ITGs (e.g., αvβ3, αvβ5, and αvβ8) are significantly upregulated in brain metastases of various solid tumors compared with primary tumors, indicating that αv ITGs can help tumor cells penetrate the blood-brain barrier and colonize the brain parenchyma.^110^^,^^126^^,^^127^ Moreover, overexpression of αv ITGs in melanoma cells can accelerate cell migration *in vitro* and facilitate the adhesion of melanoma cells to cerebral blood vessels *in vivo*, increasing the incidence of brain metastasis in athymic rat models.^128^ Further experiments suggest that αvβ3 may play a specific role in inducing brain metastasis of melanoma.^129^ Hoshino et al.^11^ also showed that β3 ITG primarily exists in encephalotropic exosomes, which can interact with CD31 brain endothelial cells (Table 2).

### ITGs and bone metastasis

ITG αvβ6, an RGD-binding protein that can bind to the TGF-β precursor peptide, can trigger EMT.^66^ Reportedly, ITG αvβ6 is associated with cancer progression and poor clinical prognosis in various tumors. Dutta et al.^130^ found that ITG αvβ6 is related to TGF-β-mediated MMP2 activation, which can initiate the osteolysis of prostate cancer, promoting bone metastasis. Fedele et al.^131^ concluded that αvβ6-containing prostate cancer exosomes immediately colonize the bone marrow by transferring to αvβ6-negative receptor cells, proving that ITG-related phenotypes can promote cell migration through horizontal transmission. Furthermore, cancer cell exosomes can transmit αvβ6 to monocytes, promote M2 polarization, and then inactivate the STAT1-MX1/2 pathway, of which STAT1 is vital for tumorigenesis and development.^132^ Additionally, αvβ3 has anti-tumor effects, including promoting M1 polarization and STAT1 activation; thus, β6 and β3 can compete with the αv subunit for binding and promote M2 polarization.^132^ However, ITG αvβ3 also exists in a cell model of advanced prostate cancer bone metastasis.^116^ Experiments have confirmed that αvβ3 on melanoma cells may also promote melanoma bone metastasis through the ERK/MAPK pathway.^133^ Furthermore, αvβ3 plays an important role in promoting tumor angiogenesis.^134^

Overexpression of ITG α4β1 on primary melanoma cells may be related to increased bone metastasis, which may be caused by interactions with VCAM-1 expressed on bone marrow stromal cells.^135^ In addition, ITG α2 is also associated with prostate cancer bone metastasis (Table 2).^136^

## Isolation and identification of exosomes

In 2015, the International Society for Extracellular Vesicles (ISEV) noted that the purity and yield of exosomes obtained simply by a single separation method would not meet experimental requirements. Therefore, a combination of methods is recommended to obtain exosomes with high purity and high yield.

Ultra-high speed centrifugation is a common, effective, and reliable method for exosome extraction. The required exosomes can be obtained at different centrifugation speeds. This method is simple to operate but time consuming, and the quantity and quality of obtained exosomes are largely affected by rotor type, the angle of rotor settlement, and other factors.^137^^,^^138^ Density gradient centrifugation uses a specific medium in the centrifugation tubes to form a density gradient, and then through a certain centrifugal force in different gradients, distinct zones are formed. Compared with ultra-high speed centrifugation, this method is more time consuming and complicated and obtains fewer exosomes. However, its advantage lies in the high purity of the obtained exosomes and the ability to maintain the original biological activity of exosomes.^138^ Both size-exclusion chromatography and ultrafiltration are methods to separate exosomes according to their size. Size-exclusion chromatography does not require much centrifugal force, thus ensuring exosome integrity.^139^^,^^140^ Ultrafiltration may deform or rupture the exosomes due to adhesion of the exosomes to the ultrafiltration membrane, but this method does not require special equipment to isolate the exosomes in a short time.^140^ Immunoaffinity chromatography, polyethylene glycol (PEG)-base precipitation, and magnetic bead-based immunoassays are also commonly used methods for extracting exosomes.^138^^,^^141^^,^^142^ Recently, with the continuous development of exosomes research, commercial test kits have become more widely used. Currently, the most commonly used kits are ExoQuick, miRCURY, and TEIR. The kit method is easy to operate, efficient, and convenient and can obtain exosomes at a high recovery rate.^143^^,^^144^ However, the obtained exosomes have many impurities, which affect their morphological characteristics under electron microscopy. Whether these extracted impurities affect the biological activities of purified exosomes will require further research.

Currently, there are four main methods of identifying exosomes: electron microscopy, nanoparticle tracking analysis (NTA), western blotting, and the polymerase chain reaction (PCR). Both ordinary transmission electron microscopy and cryo-electron microscopy are used in morphological studies of vesicles.^144^^,^^145^ Among them, cryo-electron microscopy is used to observe temperature-sensitive samples such as proteins and biological slices, as this method can reduce damage to the sample by electron beams and obtains more realistic sample morphology.^145^ NTA refers to the measurement of exosomes using the Malvin Nanoparticle Tracking Analyzer, which does not damage the structure or function of exosomes. NTA is easy to perform and can protect the structure and function of exosomes from damage. In recent years, NTA has gradually become the gold standard for identifying exosomes.^140^^,^^146^ Due to the diversity of protein components contained within exosomes and the conserved proteins that maintain exosome functions (e.g., Alix and TSG101, components of the endosome sorting and transport complex, and CD63, CD9, and CD81 of the four transmembrane protein superfamilies), western blotting is one of the most commonly used methods for detecting exosomes.^140^^,^^144^ However, PCR is the most sensitive and reliable method for detecting gene expression and thus has become an indispensable detection method in exosomal miRNA research.^147^

## Applications of exosomes for antitumor therapy

Recent studies have shown that exosomes cannot only be used as markers for the differential diagnosis of tumors but also for antitumor treatments, suggesting potential therapeutic value in a variety of tumor types.

First, exosomes are capable of delivering a variety of biomolecules and have the potential to act as natural carriers. Compared with synthetic drug carriers, exosomes have significant advantages such as stability in serum and tissues, immune escape, long circulation time, no obvious toxicities or side effects, ability to load with a variety of drugs and biomolecules, tumor cell-specific delivery, and ability to assimilate in different intracellular transport pathways. Currently, many different forms of exosomes have been developed to carry small molecule anticancer drugs. Studies have found that the use of exosomes to deliver small molecule inhibitors such as paclitaxel and doxorubicin cannot only reduce the toxicities of the drugs but also improve their *in vivo* bioavailability.^148^^,^^149^ In addition, studies have confirmed that surface modification or functional ligand modification has important significance for improving the transport performance of exosomes.^150^^,^^151^ Koh et al.^152^ found that by binding signal regulatory protein α (SIRPα) to the surface of exosomes, which then interferes with the CD47-SIRPα interaction between cancer cells and bone marrow-derived macrophages, it can enhance tumor phagocytosis and significantly inhibit tumor growth. Animal experiments by Kim et al.^153^ have also confirmed that exosomes modified by aminoacetamide (AA)-PEG can accumulate in large amounts in tumors, thereby prolonging the action time of exosomes loaded with drugs and enhancing the anti-tumor effect.

Second, several studies have confirmed that tumor-derived exosomes can present the tumor surface-specific antigens they carry to CD8^+^ T cells through DCs in the form of carriers, causing immune responses and tumor cell eradication.^154^ In addition to the tumor-associated antigens, the proteins and miRNAs contained within tumor-derived exosomes can also be used as regulatory molecules to modulate immune responses. Cho et al.^155^ found that HSP70 was abundant in tumor cell-derived exosomes, which could enhance the antigen-presenting activity of DCs, induce immune responses from T helper type 1 (Th1) cells without MHC limitations, and play an antitumor immune role. Studies on HCC-derived exosomes have found that exosomes carrying HSP70 can induce NK immune responses.^156^ This discovery provides valuable clues for the development of highly effective liver cancer immune vaccines. Currently, this model of tumor immunotherapy has entered the stage of clinical trials.

Third, because tumor-derived exosomes play important roles in tumor invasion and metastasis, removing specific tumor-derived exosomes from circulation has become a new idea for inhibiting metastasis. Aethlon Medical (San Diego, CA, USA) has designed a hemodialysis approach called Aethlon Adapt, which captures a large number of antibodies and other similar molecules, such as nucleic acid aptamers, protein ligands, and exosomes, to specifically eliminate tumor-derived exosomes.^157^

### Outlook

Exosomes are active nanoscale complexes that are required for intercellular communication. As such, they have an elaborate and diverse composition and are widely distributed in various tissues and organs throughout the body. Exosomes play an important role in the occurrence and development of various physiological activities/processes and are especially secreted by tumor cells. Tumor-derived exosomes have been confirmed to play important roles in metastasis as well as many other aspects of tumor development. Recent experiments have confirmed that RNA from tumor cells can be transferred to epididymal sperm, indicating that exosomes can be transferred to germ cells, which may lead to the cross-generational transmission of cancer-associated molecules to offspring.^158^ These properties and characteristics indicate that exosomes have great potential as natural tumor markers. Distant tumor metastasis has always been an important factor for the survival and quality of life of cancer patients. That surface ITGs of tumor-derived exosomes can locate metastatic sites at the early stage implies important roles for exosomal ITGs in this process and highlights their prospects for clinical applications. Previous studies have emphasized that ITGs may be valuable targets; therefore, precisely targeting exosomal ITGs may be a future treatment option for advanced tumors. Whether exosomes are used as drug carriers or vectors for genetic modifications to treat tumors, they have natural advantages over synthetic vectors or other treatment methods, giving them great potential as a future anticancer treatment. Therefore, further improvements in exosome purification technologies and identification methods will be required to fully harness the power of exosomes for diagnosing and treating cancers.
